# The “M. D. S.”

**Published:** 1884-02

**Authors:** C. S. Stockton

**Affiliations:** No. 15 Cedar Street, Newark, N. J.


### THE "M. D. S."

No. 15 CEDAR STREET,
NEWARK, N. J., Jan. 15, 1884.

*Editor Independent Practitioner:*

There has been some wild criticism, by some of my New York friends, concerning my remarks at Niagara in reference to the "M. D. S." law in that State. The only thing I said was that the existence of such a law in New York was made the excuse for an imitation one in New Jersey.

The bill before our legislature last year, giving authority to three men to confer a State title, was opposed, and fortunately did not become a law. It was again introduced this year, but I am just informed has been withdrawn, and another substituted. The substitute prohibits any one who is not a graduate of a reputable Dental College, from commencing the practice of dentistry in the State.

New Jersey is making progress. It is said the New York law was enacted, some fifteen years ago, for the purpose of giving some old and worthy practitioners a reputable standing. Would it not be well to complete the list, and then repeal the law, and thus place the State Society in harmony with the sentiment of the dental profession?

Very truly yours,

C. S. STOCKTON.

